# Supplementary material for: EnzML: multi-label prediction of enzyme classes using InterPro signatures
Source: BMC Bioinformatics. 2012 Apr 25;13:61. doi: 10.1186/1471-2105-13-61 (PMC3483700; doi:10.1186/1471-2105-13-61)
Supplement: Addtional file 5 — The Java code to format the data files, evaluate and predict. The file enzml_java_code.tar.gz contains the Java code used to format database data to ARFF and XML formats, to execute cross and train-test (jackknife) evaluations and to record evaluation results to database. More information is included in the readme.txt file and the Javadoc files. The code can be used with a MySQL database. To use a different database software, other JDBC drivers might be required. [file 1471-2105-13-61-S5.gz › java_code/utils/doc/uk/ac/ed/inf/utils/ArrayUtils.html]

ArrayUtils


---


|  |  |  |
| --- | --- | --- |
| **Overview** \| **Package** \| **Class** \| **Use** \| **Tree** \| **Deprecated** \| **Index** \| **Help** | |  |
| PREV CLASS   **NEXT CLASS** | **FRAMES**    **NO FRAMES**     **All Classes** |  |
| SUMMARY: NESTED \| FIELD \| CONSTR \| METHOD | DETAIL: FIELD \| CONSTR \| METHOD |  |


---


## uk.ac.ed.inf.utils Class ArrayUtils

```
java.lang.Object
  uk.ac.ed.inf.utils.ArrayUtils
```

---

``` public class ArrayUtils extends java.lang.Object ```

Utilities to manipulate arrays. Reminder: indexing of array of arrays cells >
[row index][column index]

**Version:**
:   4 Jan 2008

**Author:**
:   Luna De Ferrari luna.deferrari-at-ed.ac.uk

---

**Constructor Summary**

`ArrayUtils()`


| **Method Summary** | |
| --- | --- |
| `static boolean` | `arrayIsEmpty(java.lang.String[] array)`             Checks whether an array is empty |
| `static java.util.Vector<java.lang.String>` | `fromArrayofArraysToColumnVector(java.lang.String[][] array, int columnIndex)`             Converts a column of an array of arrays into a vector |
| `static java.util.ArrayList` | `fromArrayToArrayList(java.lang.Object[] array)`             Creates an arrayList from an array |
| `static java.util.Vector` | `fromArrayToVector(java.lang.Object[] array)`             Creates a vector from an array |
| `static java.util.ArrayList<java.lang.String>` | `fromStringArrayToArrayList(java.lang.String[] array)`             Creates an arrayList from an array |
| `static int[]` | `getCommonElements(int[] list1, int[] list2)`             Gets the elements present in both lists |
| `static java.util.LinkedHashSet` | `getCommonElements(java.lang.Object[] list1, java.lang.Object[] list2)`             Gets the elements present in both lists |
| `static java.lang.String` | `getString(java.lang.String[] array)`             Returns a string representation for an array |
| `static void` | `printArray(java.lang.String[] array)`             Prints an array (String[]) |
| `static void` | `printArrayOfArrays(java.lang.String[][] matrix)`             Prints an array of arrays (String[][]) |
| `static void` | `printArrayOfObjects(java.lang.Object[] array)`             Prints an array of objects |
| `static java.lang.String[][]` | `stringToArrayOfArrays(java.lang.String string, java.lang.String regExp)`             Reads a special-character-separated string (eg comma or tab separated) and creates an array of rows-columns. |

**Methods inherited from class java.lang.Object**

`equals, getClass, hashCode, notify, notifyAll, toString, wait, wait, wait`

**Constructor Detail**

### ArrayUtils

```
public ArrayUtils()
```


**Method Detail**

### arrayIsEmpty

```
public static boolean arrayIsEmpty(java.lang.String[] array)
```

:   Checks whether an array is empty

    :   **Parameters:**: `array` - an array of strings (String[]) **Returns:**: true if the array is empty, false otherwise

---


### fromArrayofArraysToColumnVector

```
public static java.util.Vector<java.lang.String> fromArrayofArraysToColumnVector(java.lang.String[][] array,
                                                                                 int columnIndex)
```

:   Converts a column of an array of arrays into a vector

    :   **Parameters:**: `array` - an array of arrays: `columnIndex` - the column index (the first column index is 0) **Returns:**: a vector containing the first column

---


### fromArrayToArrayList

```
public static java.util.ArrayList fromArrayToArrayList(java.lang.Object[] array)
```

:   Creates an arrayList from an array

    :   **Parameters:**: `array` - a Object[] **Returns:**: an ArrayList

---


### fromArrayToVector

```
public static java.util.Vector fromArrayToVector(java.lang.Object[] array)
```

:   Creates a vector from an array

    :   **Parameters:**: `array` - a Object[] **Returns:**: a vector

---


### fromStringArrayToArrayList

```
public static java.util.ArrayList<java.lang.String> fromStringArrayToArrayList(java.lang.String[] array)
```

:   Creates an arrayList from an array

    :   **Parameters:**: `array` - a String[] **Returns:**: an ArrayList

---


### getCommonElements

```
public static int[] getCommonElements(int[] list1,
                                      int[] list2)
```

:   Gets the elements present in both lists

    :   **Parameters:**: `list1` - a list: `list2` - another list **Returns:**: the set containing the common elements (each element only once)

---


### getCommonElements

```
public static java.util.LinkedHashSet getCommonElements(java.lang.Object[] list1,
                                                        java.lang.Object[] list2)
```

:   Gets the elements present in both lists

    :   **Parameters:**: `list1` - a list: `list2` - another list **Returns:**: the set containing the common elements (each element only once)

---


### getString

```
public static java.lang.String getString(java.lang.String[] array)
```

:   Returns a string representation for an array

    :   **Parameters:**: `array` - the String array **Returns:**: a string representation for the array

---


### printArray

```
public static void printArray(java.lang.String[] array)
```

:   Prints an array (String[])

    :   **Parameters:**: `array` - the array to be printed (String[])

---


### printArrayOfArrays

```
public static void printArrayOfArrays(java.lang.String[][] matrix)
```

:   Prints an array of arrays (String[][])

    :   **Parameters:**: `matrix` - the 2 dimentional array to be printed (String[row][column])

---


### printArrayOfObjects

```
public static void printArrayOfObjects(java.lang.Object[] array)
```

:   Prints an array of objects

    :   **Parameters:**: `array` -

---


### stringToArrayOfArrays

```
public static java.lang.String[][] stringToArrayOfArrays(java.lang.String string,
                                                         java.lang.String regExp)
```

:   Reads a special-character-separated string (eg comma or tab separated)
    and creates an array of rows-columns. Each column contains the column
    data elements Eg. for reading emboss fuzzpro format: SeqName Start End
    Score Strand Pattern\_name Mismatch DHET\_GLUOX 214 215 2 + AE . DHET\_GLUOX
    227 228 2 + AE .

    :   **Parameters:**: `string` - the string to be read: `regExp` - the regular expression separating the tokens in each line (e.g. a comma for comma separated values files) **Returns:**: an array of arrays in which each element is a token from the string (row by columns)


---


|  |  |  |
| --- | --- | --- |
| **Overview** \| **Package** \| **Class** \| **Use** \| **Tree** \| **Deprecated** \| **Index** \| **Help** | |  |
| PREV CLASS   **NEXT CLASS** | **FRAMES**    **NO FRAMES**     **All Classes** |  |
| SUMMARY: NESTED \| FIELD \| CONSTR \| METHOD | DETAIL: FIELD \| CONSTR \| METHOD |  |


---
